# Patient-facing job role is associated with SARS-CoV-2 positivity among healthcare workers in long term care facilities in Minnesota, August–December, 2020

**DOI:** 10.1017/ice.2022.289

**Published:** 2023-09

**Authors:** R. Adetunji Bakare, John F. Mulcahy, Matthew F. Pullen, Ryan T. Demmer, Sara L. Cox, Julie A. Thurn, Alison L. Galdys

**Affiliations:** 1 University of Minnesota School of Public Health, Minneapolis, Minnesota; 2 University of Minnesota School of Medicine, Minneapolis, Minnesota; 3 M Health Fairview Health System, Minneapolis, Minnesota

## Abstract

**Objective:**

Healthcare workers (HCWs) in long-term care facilities (LTCFs) are disproportionately affected by severe acute respiratory coronavirus virus 2 (SARS-CoV-2), the virus that causes coronavirus disease 2019 (COVID-19). To characterize factors associated with SARS-CoV-2 positivity among LTCF HCWs, we performed a retrospective cohort study among HCWs in 32 LTCFs in the Minneapolis–St Paul region.

**Methods:**

We analyzed the outcome of SARS-CoV-2 polymerase chain reaction (PCR) positivity among LTCF HCWs during weeks 34–52 of 2020. LTCF and HCW-level characteristics, including facility size, facility risk score for resident-HCW contact, and resident-facing job role, were modeled in univariable and multivariable generalized linear regressions to determine their association with SARS-CoV-2 positivity.

**Results:**

Between weeks 34 and 52, 440 (20.7%) of 2,130 unique HCWs tested positive for SARS-CoV-2 at least once. In the univariable model, non–resident-facing HCWs had lower odds of infection (odds ratio [OR], 0.50; 95% confidence interval [CI], 0.36–0.70). In the multivariable model, the odds remained lower for non–resident-facing HCW (OR, 0.50; 95% CI, 0.36–0.71), and those in medium- versus low-risk facilities experienced higher odds of testing positive for SARS-CoV-2 (OR, 1.47; 95% CI, 1.08–2.02).

**Conclusions:**

Our findings suggest that COVID-19 cases are related to contact between HCW and residents in LTCFs. This association should be considered when formulating infection prevention and control policies to mitigate the spread of SARS-CoV-2 in LTCFs.

Residents of long-term care facilities (LTCFs) have experienced disproportionately high morbidity and mortality during the coronavirus disease 2019 (COVID-19) pandemic.^1,2^ Surveillance testing for early detection of severe acute respiratory coronavirus virus 2 (SARS-CoV-2), the etiologic agent of COVID-19, is important in mitigating the spread of infection in LTCFs.^3–6^ Various factors have a documented association with COVID-19 in healthcare settings, including geographical setting,^7^ facility size,^8^ and community incidence.^9^ In this study, we assessed individual and facility-level characteristics associated with SARS-CoV-2 positivity among LTCF healthcare workers (HCWs) in a surveillance testing program.

## Methods

### Study population

This retrospective cohort study included HCWs from 32 LTCFs within a single Minneapolis–St. Paul (MSP) area healthcare system who underwent surveillance testing for SARS-CoV-2. Across the 32 LTCF sites, a range of care is provided: skilled nursing care, memory care, transitional care and/or short-term rehabilitation for persons transitioning to home after a hospital stay, and assistance for independently living older adults. The study period was March 1, 2020, to December 21, 2020.

### SARS-CoV-2 surveillance

Passive surveillance SARS-CoV-2 testing, defined as testing that occurred in individuals with symptoms compatible with COVID-19, was performed for HCWs and LTCF residents throughout the study period. Active surveillance testing, defined as testing irrespective of COVID-19 symptoms, was introduced in June 2020. All tests were reverse-transcriptase polymerase chain reaction (RT-PCR) assays of nasopharyngeal specimens for SARS-CoV-2 RNA. An initial point-prevalence survey (PPS) of all HCWs and residents at study sites was conducted in June 2020 (weeks 23–26). From June to August 2020 (ie, weeks 23–36), the active surveillance testing goal for LTCFs was set at capturing 20% of HCWs weekly. In September 2020 (week 37 onward), active surveillance was dictated by the 14-day test-positivity rate in the geographic county of each LTCF.^10^ A positivity rate <5% required that LTCF HCWs test once monthly; a rate of 5%–10% required once weekly testing; and a rate >10% required twice weekly testing. The source of data for the 14-day county positivity rate was the Centers for Medicare and Medicaid Services (CMS) website, which publicized county-specific SARS-CoV-2 test-positivity rates.^11^ Detection of SARS-CoV-2 in an LTCF prompted a facility-wide PPS. Facility PPSs were repeated every 3–7 days until 14 days passed since the last potential exposure to a SARS-CoV-2–positive individual. Individuals who tested positive were not retested for 90 days based on public health recommendations.^16^ We collected individual-level data within the Occupational Health and Safety Management (OHM (UL, Northbrook, IL) electronic record, into which data on SARS-CoV-2 testing was manually entered.

### Outcomes

The outcome was positive RT-PCR for SARS-CoV-2 RNA reported at the individual level.

### Covariables

HCW covariables included job role, work site, and testing results. Facility covariables included facility type, facility size, and facility risk score. Facility type was dichotomized into assisted living–independent living and skilled nursing facility. Resident-facing status was defined as “yes” for HCWs whose job roles entailed direct resident contact and “no” for those whose job roles did not entail direct resident contact (Supplementary Material online). A COVID-19–dedicated unit was opened during week 48; activities on this unit are not captured in our covariables. LTCFs were categorized into quartiles based on the combined number of residents and HCWs: (1) smallest (100–150 residents), small (151–183 residents), moderate (184–223 residents), and large (224–415 residents). LTCF personnel categorized the facility risk score for resident-to-HCW contact as highest risk, medium risk, and lowest risk. Facility risk was proportionate to the skilled needs of residents; the highest-risk facilities housed the largest number of residents occupying memory care beds or who depended on HCWs for activities of daily living. The multivariable regression analysis was also adjusted for weekly county-level SARS-CoV-2 positivity data, which were retrieved from the CMS website.

### Statistical analysis

Descriptive information concerning numbers tested, numbers diagnosed, and proportion positive were assessed over the study period: March 1, 2020, to December 21, 2020. Our multivariable analysis comprised a shorter study period, from August 17, 2020, to December 21, 2020, because SARS-CoV-2 community prevalence data from CMS were only available beginning August 17, 2020. Two analyses were conducted. The first analysis assessed infection risk factors at the individual level with emphasis on individual level risk factors. Logistic regression models were utilized in SAS proc genmod software. Participants were conditioned as random effects to account for repeated measures or clustering by facility. A second analysis was performed at the facility level and assessed facility-level factors predicting overall cumulative incidence within facilities. County SARS-CoV-2 prevalence for each facility was time varying and was treated as a continuous variable. These analyses were conducted using generalized linear regression models. Data were cleaned and analyzed with SAS version 9.4 software (SAS Institute, Cary, NC).

### Ethics approval

Ethical approval was obtained from the University of Minnesota Institutional Review Board.

## Results

**Fig. 1.**
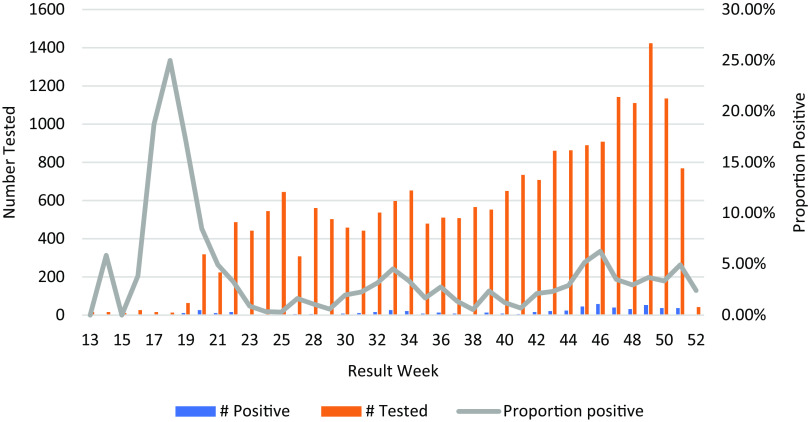
Distribution pattern of the total number of tests, total positives, and overall proportion positive among healthcare workers tested for SARS-CoV-2 in 32 long-term care facilities, March–December 2020.

**Fig. 2.**
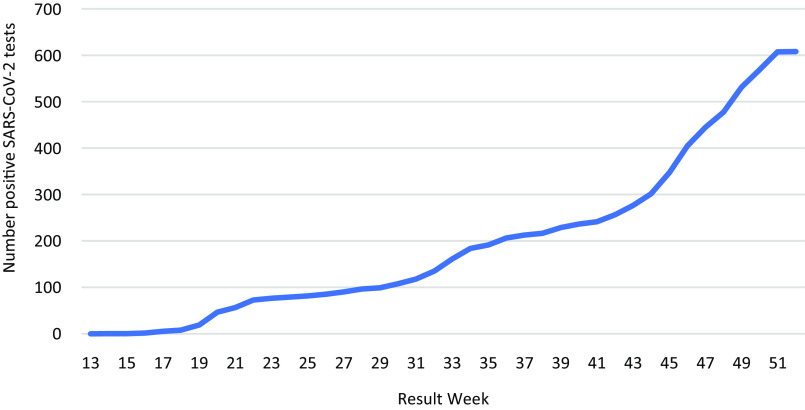
Cumulative number of positive SARS-CoV-2 tests among healthcare workers in 32 long-term care facilities over time, March–December 2020.

SARS-CoV-2 test positivity peaked in week 18 at 25% and subsequently ranged from 0.31% to 6.3% between weeks 20 and 52 (Fig. 1). The cumulative number of positive SARS-CoV-2 tests from March to December 2020 was 608 (Fig. 2). Most of the LTCFs in the sample were assisted living or independent living facilities and were characterized as having the lowest exposure risk (Table 1).

**Table 1.** Long-Term Care Facility Characteristics

Variable	No. (%)
Total facilities	32
**Facility size characterization (total HCWs and residents)**	
Smallest (100–150)	8
Small (151–183)	8
Moderate (184–223)	8
Large (224–415)	8
**Facility type**	
Assisted living/independent living	28 (87.5)
Skilled nursing facility	4 (12.5)
**Facility risk of resident to HCW exposure score**	
Highest risk	4 (12.5)
Medium risk	5 (15.63)
Lowest risk	23 (71.88)

Note. HCW healthcare worker.

During weeks 34–52 (August 17, 2020–December 21, 2020), 14,526 tests were obtained from 2,130 HCWs, 445 of which were positive (Table 2). Moreover, 5 HCWs tested positive on 2 occasions 90 days apart. Therefore, 440 (20.66%) of 2,130 unique HCWs tested positive at least once. The median number of tests for non–resident-facing HCWs was 8 (interquartile range [IQR], 6), and for resident-facing HCWs it was 6 (IQR, 6). The median number of tests among participants with all negative results was 6 (IQR,7). Most HCWs worked in close contact with the residents.

**Table 2.** SARS-CoV-2 Test Positivity and Test Frequency Characteristics Among HCWs During Sampling Period Encompassing Weeks 34–52 of 2020

Variable	No. (%)	SARS-CoV-2 Positive, No. (%)
Resident-facing HCW	1,910 (89.67)	409 (21.41)
Non–resident-facing HCW	220 (9.46)	36 (16.36)
**HCW work site**		
Smallest	339 (15.9)	57 (16.81)
Small	427 (20.1)	74 (17.33)
Moderate	488 (22.9)	85 (17.42)
Large	876 (40.1)	229 (26.14)
HCW PCR tests, no.	14,526	
Positive PCR results, no.	445	
Tests per resident-facing HCW, median (IQR)	6 (6)	
Tests per non–resident-facing HCW, median (IQR)	8 (6)	
Tests among negatives, median (IQR)	6 (7)	

Note. HCW, healthcare worker; IQR, interquartile range; PCR, polymerase chain reaction.

Our univariable analysis shows that resident-facing status was associated with higher odds of SARS-CoV-2 positivity (Table 3). HCWs whose job roles did not entail direct contact with residents had a lower odds of SARS-CoV-2 positivity compared to HCWs with direct resident contact (odds ratio [OR], 0.50; 95% confidence interval [CI], 0.36–0.70; *P* < .0001). The association between facility risk score and SARS-CoV-2 positivity was not statistically significant in the univariable regression (*P* = .25).

**Table 3.** Univariable and Multivariable Predictors of SARS-CoV-2 Positivity Among 2,130 LTCF HCW During Weeks 34–52 of 2020

Variable	Univariable		Multivariable^a^	
	OR (95% CI)	*P* Value	OR (95% CI)	*P* Value
**Resident facing**		<.0001		<.0001
Yes	Reference		Reference	
No	0.50 (0.36–0.70)	<.0001	0.50 (0.36–0.71)	<.0001
**Facility size**		.8997		.9211
Smallest	0.99 (0.73–1.32)	.9223	1.12 (0.71–1.76)	.6346
Small	0.91 (0.69–1.20)	.5017	1.06 (0.69–1.63)	.7899
Moderate	1.02 (0.79–1.32)	.8753	1.14 (0.75–1.75)	.5309
Large	Reference		Reference	
**Facility risk of resident to HCW exposure score**		.2497		.0730
Highest risk	1.10 (0.90–1.35)	.3550	1.29 (0.85–1.94)	.2269
Medium risk	1.29 (0.96–1.74)	.0919	1.47 (1.08–2.02)	.0156
Lowest risk	Reference		Reference	
County positivity rate	1.69 (1.68–1.70)		1.12 (1.09–1.15)	<.0001

Note. LTCF, long-term care facility; HCW, healthcare worker; OR, odds ratio; CI, confidence interval.

a Multivariable results are presented, based on a model that includes all the variables in the table.

In multivariable models, non–resident-facing HCW roles were associated with lower odds of a positive test (OR, 0.50; 95% CI, 0.36–0.71; *P* < .0001), and county positivity rate was associated with higher odds of a positive tests result (OR, 1.12; 95% CI, 1.09–1.15; *P* < .0001). Although facility risk factors did not have any overall effect on risk of infection, working in a facility with medium risk compared to those with lower risk was associated with increased risk of positivity (OR, 1.47; 95% CI, 1.08–2.02; *P* = .02) (Table 3).

## Discussion

In this retrospective cohort study of HCWs in 32 LTCFs in an urban area, the risk of SARS-CoV-2 positivity was associated with community positivity rates and with resident-facing job roles.

Published data on the association between SARS-CoV-2 positivity and HCW role are somewhat mixed. Although several investigations have identified SARS-CoV-2 seropositivity risk among patient-facing versus non–patient-facing HCW roles in LTCFs,^12,13^ other investigations sampling heterogenous HCW settings have not demonstrated an association between SARS-CoV-2 positivity and HCW role.^9,14^ One explanation for this is the direct interaction between HCWs and residents in LTCFs, such as during toileting, feeding, and other activities of daily living (ADLs) may be unique to LTC settings and distinct from the patient-to-HCW interactions in acute-care settings.^7,15^ An additional explanation for the association between LTCF job role and COVID-19 infection is that personal protective equipment (PPE) use, which has been demonstrated as effective in mitigating the risk of COVID-19 transmission in HCWs,^16^ is challenged in LTC settings. A recent investigation in Minnesota found that significantly fewer HCWs used PPE during exposure to residents with COVID-19.^17^ Appropriate use of PPE requires both an adequate supply and knowledge of proper use. Due to inadequate PPE supplies and high HCW turnover,^18^ HCWs in LTCFs often did not have PPE available, and even when PPE was available, a lack of proficiency with PPE diminished its potential effectiveness.

This study had several limitations. Our study sample lacked HCW demographic data. Variables related to social determinants of health were unmeasured in our sample and may be a source of confounding. Importantly, black race^12,14^ and Hispanic ethnicity^12^ have been associated with SARS-CoV-2 seropositivity in HCWs. In larger samples of county-level data, COVID-19 risk has been associated with not only race and ethnicity affiliation but also income inequality, education deprivation, and underinvestment in infrastructure.^19–21^ In addition, although we controlled for community positivity, we were unable to assess known occupational or household COVID-19 in our sample, and these variables are known to influence SARS-CoV-2 positivity in HCWs.^9,12^ Furthermore, although the COVID-19–dedicated unit in our sample was not opened until week 48 of 52, we did not assess its impact on SARS-CoV-2 positivity among HCWs, which is a limitation given prior work demonstrating higher risk in HCWs on COVID-19–dedicated units in LTCFs.^12^

Our study was not designed to ascertain the directionality of SARS-CoV-2 transmission in LTCFs. We hypothesized that SARS-CoV-2 entry into LTCFs occurred principally via infected HCWs, based on our presumption that visitor restrictions in place at the time of our investigation served as a substantial barrier to transmission from non-HCWs. Following the introduction of SARS-CoV-2 into a facility, both residents and HCWs become reservoirs for transmission.

Although recent studies have reported that larger facility size was consistently associated with increased risk of COVID-19 infections and outbreaks,^8,22^ we found that facility size was not associated with the risk of COVID-19 infection. Other investigations have identified facility staffing levels,^23^ facility profit status,^24^ and staff-to-resident ratio^25,26^ as being associated with SARS-CoV-2 positivity. These variables were not available for inclusion in our analysis and may be other sources of confounding in our analysis.

The strengths of our study include the large size of our sample and the large number of tests performed. In addition to those previously mentioned, this study had several limitations. We calculated odds ratios for our outcomes of interest, and we acknowledge that these estimates may differ substantially from risk ratios in our sample given the high number of observed outcomes. Our results cannot be generalized to other congregate settings because our investigation was limited to the subpopulations of HCWs in LTCFs in the Minneapolis–St. Paul area. The findings from our urban sample may not be generalizable to LTCFs in rural areas.^22^ Facility size characterization was based on capacity and not actual census, so it may not accurately represent the numbers of residents and HCWs. SARS-CoV-2 testing by PCR of nasopharyngeal swab samples was not compulsory or universal; thus, we may have missed convalescent infection. Possibly, testing was biased toward a subset of HCWs. Our study period occurred prior to vaccine availability for COVID-19; thus, we did not consider the vaccination status of HCW and residents.

We did not assess the impact of active surveillance SARS-CoV-2 testing on the positivity trends in LTCFs. Based on the sizeable proportion of persons who transmit SARS-CoV-2 asymptomatically,^27^ we hypothesize that universal testing of HCWs in LTCFs may be an important component of infection control policies to mitigate SARS-CoV-2 transmission. This hypothesis warrants further study.

In conclusion, we observed that HCWs with job roles involving direct resident contact in LTCFs are at higher risk for COVID-19 than HCWs whose job roles at LTCF do not involve resident contact. This finding should inform COVID-19 mitigation strategies at LTCFs, including policies related to PPE.

## Supplementary material

For supplementary material accompanying this paper visit https://doi.org/10.1017/ice.2022.289. click here to view supplementary material
