# Supplementary material for: Patient-facing job role is associated with SARS-CoV-2 positivity among healthcare workers in long term care facilities in Minnesota, August–December, 2020
Source: Infect Control Hosp Epidemiol. 2023 Mar 13;44(9):1467–71. doi: 10.1017/ice.2022.289 (PMC10507513; doi:10.1017/ice.2022.289)
Supplement: Supplementary file 1 [file S0899823X22002896sup001.docx]

Supplementary Data

Table 1. Twenty-five job categories were created by study personnel based on 288 unique, free-text job titles for sampled long-term care facility healthcare workers. Each job category was designated as resident facing (or non-resident-facing) by study personnel.

| Job category | Resident-facing (y/n) |
| --- | --- |
| Allied patient care staff | y |
| Administration | n |
| Admissions coordination | n |
| Adult Daycare | y |
| Child care | y |
| Cooking staff | n |
| Nursing | y |
| Finance | n |
| Nutrition | y |
| Chaplain | y |
| Staff Education | n |
| Facilities | y |
| Information Technology | n |
| Dietician | y |
| Environmental Services | y |
| Rehabilitation | y |
| Social Services | y |
| Sign Language | y |
| Volunteer services | n |
| Human Resources | n |
| Emergency Medical Services | y |
| Home Care | y |
| Infection Prevention | n |
| Occupational Therapy | y |
| Physical Therapy | y |
